# New Appliances and Things Medical

**Published:** 1903-06-13

**Authors:** 


### NEW APPLIANCES AND THINGS MEDICAL.

[We shall be glad to receive at our Office, 28 & 29 Southampton Street, Strand, London, W.C., from the manufacturers, specimens of all new preparations and appliances which may be brought out from time to time.]

### THE ALLENBURY BABY FOODS.

(Allen and Hanburys, Limited, Bethnal Green, London, E.)

We are glad to notice that the system of feeding babies, known as the Allenbury, has recently been modified in such a measure as to conform to the conditions which are laid down by those who make this branch of dietetics a special study. The modifications which have been introduced for the most part concern the exact percentage composition of the foods during the different periods of infancy, and the directions which are given as regards the quantity to be administered at one feeding. The Allenbury foods, Nos. 1, 2, and 3, are chemically constituted so as to form a carefully-adjusted and progressive series suitable to the developing capabilities of the infant's digestion. To assist both the doctor and the nurse in the preparation of foods of percentage constitution suitable for premature and delicate infants some very useful formulæ are printed on special cards. It is thus possible for those who make use of the Allenbury foods to meet all contingencies in abnormal cases, and in normal cases to supply a scientifically adapted food, by following the directions which are supplied by the manufacturers. In conclusion, we would suggest that as regards the proportion of sugar the 10 per cent. basis which is adopted in the Allenbury foods is too high for ordinary purposes; 6 per cent. would probably give as good or even better results.

